# The Role of Technology-Based Education and Teacher Professional Development in English as a Foreign Language Classes

**DOI:** 10.3389/fpsyg.2022.910315

**Published:** 2022-06-10

**Authors:** Weihong Zhang

**Affiliations:** Department of Foreign Languages, Zhengzhou Business University, Gongyi, China

**Keywords:** EFL classes, teacher professional development, technology-based education, technology-based instruction, educational activities

## Abstract

The swift development of technology has had a considerable effect on teaching, especially in foreign language classes, and the rising procedure of using creative technology to help teachers’ instruction and learning indicates the growing domination of technology in academic environments. In addition, teacher professional development significantly affects enhancing the teaching quality, especially the quality of educational activities within the class. Nevertheless, the shortage of workshops on professional development education made educators reliant on informal education where they worked and learned collectively with classmates in mini-groups to enhance their technology usage. The functions of technology-based instruction in the process of learning have not been taken into account in the professional development programs in the Chinese context so far, and consequently, this review takes a look at this issue. In a nutshell, this review of literature has suggestions for academics, theoreticians, and experts in search of inspecting the roles of technology in teacher professional development programs.

## Introduction

At present, language learning and education are considered as an attractive topic worldwide and, for decades, English education and learning have been experiencing numerous demanding situations and issues, though the vital function of English as a Foreign Language (EFL) educators is indicated by designing an effective curriculum initiative that is beneficial to succeed when facing difficulties (Jiang et al., 2019). One of the most efficient methods to strengthen educators is assumed to be professional development, which is a lifelong effort, a method of being, and a viewpoint on how individuals practice (Hartono, 2016) and such procedure in education means educator growth, and it is also mentioned that stable learning is vital to educator growth (Wong, 2011). Essentially, professional development refers to teachers’ education, adapting the method of learning, and conveying their information into practice that learners can use to develop themselves (Avalos, 2011). Many policy-makers, practitioners, and scholars in the education realm are unanimous that educator professional development will truly enhance learning outcomes of learners (Tajeddin and Rezanejad, 2019). The quality of teacher professional development is turning into a highly important academic matter, as educators come across increasing investigation and stress to assist learners to reach higher degrees. Although discussion and stresses over the field shape and concentrate on teacher professional development, educators are anticipated to fulfill based on new and altering criteria, and school areas are pushing educators to reform activities through learning tasks ranging from conference, workshop to class modeling (Margolis et al., 2017). Professional development is crucial in assuring educators to maintain developments in complete learner fulfillment criteria, learn new education approaches within the content domains, discover ways to learn educational modern technologies for education and learning in the best way, and making their education adaptive to the changing school setting and an increasingly different learner population (Lawless and Pellegrino, 2007).

The progress of technology has provided teachers with extensive access to learning and professional development without having time and space boundaries (Kao et al., 2014). Indeed, using technology for educator improvement builds affinity spaces that vary from conventional environments confined by bureaucracies and hierarchies (Gee, 2004). Undeniably, because of the advent of technology, the English language education method has been substantially modified. Technology provides many advantages such as making education fascinating and more effective regarding improvements. Using technology facilitates students’ engagement and learning based on their favorites. This issue is widely admitted to English education in the modern world. Technology meets the student’s audiovisual feelings (Shyamlee and Phil, 2012). Technology has become a part of our normal life (Aghaei et al., 2020); therefore, it turns to reconsider the notion of merging technology into the educational program and focusing on its integration into education to help the learning procedure. That is, technology turns into an essential component of the learning experience and an important issue for educators, from the start of making the learning experience ready to the process of learning and instruction (Altun and Khurshid Ahmad, 2021). Regarding preparation of the learners for the present digital technology, educators are considered as the main actors in the use of technology in classes every day, i.e., due to its capability in presenting a lively and proactive education-learning setting (Arnseth and Hatlevik, 2010). Although technology merging aims at enhancing and growing the quality, availability, and cost-effectiveness of instructing learners, it also mentions the benefits of networking groups of learning to manage the demanding situations of present-day globalization (Albirini, 2006; Yunus, 2007). Its adoption procedure is not done in a single step (Lee and James, 2018); however, it has consistent and non-stop steps that assist education and learning and knowledge sources (Young, 2003). Technology plays a significant function in improving tasks for students and has a substantial impact on educators’ teaching approaches. If educators fail to use technologies in their education, they will fail to keep pace with such methods. Therefore, educators need to have complete information about those technologies when instructing language competencies (Gilakjani, 2017).

As stated by Yadav et al. (2018), the recent years of the twenty-first century witnessed a vast and large number of studies and results in the area of merging technology to learning schemata as a firm learning instrument. The emergence of the digital era and the significance of merging new technology have substantially modified the learning and communicating manner. The social effect of technology is incomprehensible because the popularity and the wide use of the Internet after its discovery caused significant evolutions in our community (Martins et al., 2016). Additionally, within the present state of the COVID-19 pandemic, merging technology into online language education has (unavoidably) obtained much attention. Disregarding the obstacles to merging technology into language education and learning, the COVID-19 pandemic accelerated the technology merging process (Aghaei et al., 2022). Indeed, modern technology is merged in the world of teaching on a large scale to help the learning process in numerous fields such as English (Adnan et al., 2019). Furthermore, technology may enhance learners’ involvement. Currently, technology is progressing at a rate that traditional education and learning methods cannot help learners and educators reach their complete capability. Through the use of technology, one can improve education and learning and provide new aspects of the same (Patil, 2020). It can be the most effective method to learn a language, particularly within crucial conditions. Preparation is thus critical for the current situation considering the information, abilities, and resources required for merging the technology (Hakim, 2020). Teachers in the EFL setting have been significantly emphasizing merging technology. Academic organizations around the world are promoting the usage of merged academic technologies inside the teaching program (Culp et al., 2005). Technology merging has become effective with the advent of technological gear in academic fields. The extensive resources and chances that computers and the Internet present have caused new methods, tactics, and instruments in the processes of language education and learning. Educators have an extensive scope of PC/cellphone apps and learning control systems accessible to them. Technology merging is not an opportunity, but a necessary one for the educators; therefore, having lower rates of willingness to use technology can only worsen the condition (Cowan, 2008). At present, virtual native learners need to learn through technology instead of traditional methods of language education, because technology is more convenient, more joyful, and allows more freedom to control the learning content (Ahmed, 2012).

Scholars declared that merging technology in teaching can be a method for adding to the effectiveness of educators’ teaching and learners’ learning (Cuban, 2009). In modern technology, teaching is firmly advocated by technology-permeated learning, as the use of academic technology remains within the class education. Research claims that educators who combine academic technology with in-class instruction have a deep effect on their education efficiency and learner success, particularly in instructing English students (Jabbari et al., 2017). In line with the review of literature, English language educational program is prevalently executing technologies with the purpose to enhance the field of education and learning and solve the likely demanding situations (Adnan et al., 2019). Numerous scholars have done research trying to figure out if embedding technology within the class aids learners and educators in this field. The impact of employing technology on teaching is investigated by various scholars in numerous fields and they are unanimous that technology enables educators to enhance their education methods and students to boost their understanding (Timucin, 2006; Gilakjani and Sabouri, 2014). Educators start using the Internet for educational functions and such use has become constant in the final stage of professional development program (Giordano, 2008). In a study, Brinkerhoff (2006) assessed the impacts of a protracted professional development academy on technology abilities, PC self-efficacy, and technology merging opinions and activities.

Based on the literature review, the teachers indicated an enhancement in their technical abilities due to their experience in the academy, which had less fear and greater confidence regarding technology and manipulated that the academy had changed their education levels. Briefly, it was found that the technology utilization capabilities of educators were enhanced, and they merged technology with learning settings after completing the professional development program (Lavonen et al., 2006). These types of programs are enhancing computer capabilities among educators, although merging technology into educational programs continues to be at a constrained stage (Yurdakul et al., 2010). Regardless of the constructive attitudes toward using technology in language learning, teachers hesitate to employ technology in the classroom due to its technical difficulties on the one hand and lack of progressive technical skills and confidence in using technology on the other hand. Moreover, based on the researcher’s knowledge, there is a paucity of an inclusive professional development training syllabus associated with technology integration in China. Indeed, they were not provided with adequate professional development training courses related to technology and they were not certain about using technology academically and theoretically. Although there are numerous studies on the practice of technology, based on the researcher’s knowledge, there is a dearth of professional development training related to technology integration in China.

## Review of the Literature

### Technology and Its Benefits

Various researchers have described technology, as it refers to practically employing information, in particular, in a determined field, which is a method of conducting a mission, in particular, the use of technical procedures, approaches, or information (Isman, 2012). Using technology consists of devices (PC hardware) and tools as well as organized relations with different human beings, devices, and the setting (Isman, 2012; Aghaei et al., 2020). According to Gilakjani (2017), the definition of technology merging refers to the methods that educators employ technology to do familiar tasks more efficaciously and how such utilization can reform those tasks. Technology merging is using technology to enhance the academic setting (Dockstader, 2008). It helps the class education through developing possibilities for students to finish tasks on the PC instead of the typical paper and pen.

Using technology in education is of higher importance in current times, as educators also need to keep pace with the technological information of their learners (Richards, 2014) to satisfy the demands of the current digital natives, who are rather skillful, and in a manner, reliant on computers and other Internet-based tools (Prensky, 2008). Moreover, using technology for education, practicing, evaluating, and learning a foreign language has many benefits, especially in EFL settings in which students have limited chances to practice and evaluate their language competencies (Alsied and Pathan, 2013). Using technology in academic tasks also has a vital function for learners’ learning engagement (Günüç and Kuzu, 2014). Therefore, technology presents possibilities to encourage learners and valid linguistic entry besides opportunities of using the language with a real conversation goal. Educators have begun describing the task types they are ready for their lessons and improve their learners’ incentive successfully through introducing tasks that are well-prepared and organized into the L2 class (Lin, 2009). Along with the Internet development, communication through computers appeared to facilitate tutors’ communication compared with traditional in-person communication. Computer-assisted communication is a tool that is globally accessible for educators to debate and think about their education tactics electronically to improve professional development (Hough et al., 2004). This issue can be confirmed by the conclusion that “technology expands and reshapes how societies prepare and state borders and relations, which modifies the engagement, peripherality, and legality dynamics” (Wenger et al., 2009).

Using technology in language learning classes can be useful for the educators and the students, as it is argued that using technology-based English language education tasks enhances collaborative language learning and allows them to proficiently apply language in communication (Harmer, 2007). Tasks based on technology present language students with a proper and suitable way of language learning in the language class. Computer tasks assist language students to access data and valid content rapidly, although the consistency of using the Internet encourages students for more learning (Gençlter, 2015). According to Freeman and Anderson (2011), technology makes education easy and offers educators proper education resources, presenting language learning experiences to the students’ world. Technology can provide students with valid knowledge. Technology is employed as a crucial and vital part of education and learning experience; it maintains important matters for the educators since the start of the final stage of education and learning procedure (Wang, 2017; Altun and Khurshid Ahmad, 2021). The approaches to language education have been modified because of the technology development and educator need to have proper information about the technology with the language education attitude (Shyamlee and Phil, 2012). Language education and learning approaches are altered due to signs of progress in technology (Gilakjani, 2017).

### Professional Developments

The phenomenon of professional development plays a significant role for educators to make progress in their job professionally. Except for their professional lives, gaining the required understanding, competencies, and educational activities should also address learners’ demands (Lee, 2014). The construct of professional development means that all varieties of professional learning committed by in-service educators of the English language are past the primary point of formal educator readiness. Educator professional development is described as a long-lasting procedure of development that contains learning with collaboration and/or independence … educators are involved in the procedure, and they think actively about their work (Crandall, 2000). The term L2 language educator training is used to mean professional readiness and continued educators’ professional development (Freeman, 2004). However, it is referred to as a wide extension of tasks made to help the educators who finished their primary education (Craft, 2000). Educators’ learning processes are initiated by educator training and professional development programs, which results in educators’ learning results. When educators master such expertise, activities, etc., in their education, they shape a vital factor of the learning milieu for the learners along with the learning content, physical setting, classmates, etc. (Krolak-Schwerdt et al., 2014).

Given that professional development is vital for reforming foreign language education, the assigned learning activities should be tailored to professional activities. The professional development program with activities according to lesson plan achievements and content is critical to set up stability in classroom practices. It offers educators a related program consistent with the curriculum perspective and material (Nicholas and Ng, 2012). Accordingly, educators may notice how to use their professional learning tasks in their educational activities and recognize professional development applications that link their experiences and real class activities (Garet et al., 2001). According to Mizell (2010), professional development means numerous forms of instructional experiences associated with people’s work. Human beings in different jobs take part in professional development to assist them to learn and use new information and abilities that enhance career performance. Professional development has become critical for educators because of vast reform initiatives that make educators highly responsible to nurture learners who carry out proper standard exams with high stakes.

Educators’ professional development complies with various methods and tactics. As an instance, the concept of collaborative educator development was promoted as something educators could perform with coworkers, college scholars, learners, or others engaged in learning and education (Johnston, 2009). Regarding professional development methods, educators can select between two general alternatives, namely, informal and formal (Wilden and Porsch, 2017). The formal method encompasses educators participating in an education plan, which fulfills a fixed educational program on a certain subject matter. Informal learning is commonly initiated and controlled by oneself. Study participants are selected due to the fact that they may be attractive to educators; consequently, educational experiences or informal communication is much better with practitioners with a higher experience (Bennett, 2012). The formal method of professional development may consequently be considered top-down; however, the informal method can be considered bottom-up.

## Implications and Future Directions

Professional development program aimed to help the teachers increase their skills and information in handling and regulating their classes and obtain a constructive attitude toward professional training and classroom upshots. At present, the importance of technology merging in academic areas is popular, as it assists students and educators in studying course content simply because of easy access (Sabzian et al., 2013). Using technology in the university, college, and school curriculum aids them in comprehending the topics well and clearing their fundamentals. Enhancements in technology significantly affect students in using what they are taught in any topic to figure their status in a universal workforce. Technology helps students’ learning and is used as the actual academic organization allowing learning to take place (Rodinadze and Zarbazoia, 2012). The application of technology in academia is highly advantageous for educators. Educators currently can cooperate to build greater meaningful education for all students without having to order planning time. Educators may employ websites to provide students with the needs and samples; however, they could not employ them as they hoped, as they had constrained information and competencies to use the technology in their class. Professional development helps educators build the meaning of technology merging in education to adjust its effect on education.

Regarding the advantages of merging technology in English language teaching (ELT) education, EFL educators need to employ technology with higher effectiveness to help their education and aid learners’ learning. Educators need innovation in the usage of academic technologies to satisfy learners’ needs. Schools are suggested to present helping equipment to contribute to EFL educators with continuous technology professional development about learning a language. Those efforts can provide great help and chances for EFL educators to improve their technology professional development in terms of pedagogy and practice that increase the quality of English language education. Presenting tasks that are in agreement with the axioms of efficient professional development can be an essential move toward lengthy modifications in educators’ understanding and activity. A teacher might have a significant effect on building such a setting and helping the elements that make an educator’s decision to apply technology for teaching. Indeed, educators can enhance their education quality and therefore increase learners’ learning achievement at schools by participating in professional development tasks.

Educators with successful experience in merging technology in their class have expressed taking part in professional development that assisted them to recognize how educational programs, criteria, and technology link (Penuel, 2006). Furthermore, some studies indicated that numerous EFL educators consider PC technology as a beneficial education instrument that increases methods of education by presenting learners with diverse language entry and extending learners’ learning experiences in actual and original fields. It is proven that education based on technology for EFL educators significantly affects the enhancement and efficiency of their education. For instance, educators who have taken part in the educational program had higher confidence regarding improving learners’ learning results by employing technology (Ansyari, 2015). In a professional development model, educators are involved in tasks with technology orientation, which is a major measure to improve educators’ understanding and capabilities to employ technologies to educate language. Educator training workshops are suggested to educate trainees about the significance of merging technology in EFL classes and educate them on different instructional technological instruments for application in EFL class, which helps their professional development (Murray, 2010).

Based on the literature review, it is known that if educators are provided with academic technology classes in their educator training programs, they can apply technology in their education with higher preparation. However, readiness programs fail to prepare educators to get ready for using technology effectively in their classes. Educator training programs must make pre-service educators ready to know and get aware of creative technologies. As a result, the technology experiences of pre-service educators of science need improvement by merging their academic technology classes into educator training plans. Successful integration of technology into teaching strongly depends on developing properly organized, consistent professional development programs designed with a transparent knowledge of the way educators require using technology in their class in the best manner. Educators need to be learners all their life to keep up with technology novelties in teaching. But educator readiness plans are insufficient for educators to keep pace with current modifications in teaching for the remaining of their jobs. Therefore, they require consistent professional development and help. Educators extend their technology by merging information and ideas and viewpoints through a professional development model consisting of designing and enacting lessons through designing groups, providing professional help, using certain software apps, and employing exemplary content. The pre-service educators can effectively merge technology if prepared with technological information and abilities. They can also do the same with technological information and competencies with a topic and might steer between such interconnected parts like a professional who can effortlessly go beyond the boundaries of topic, pedagogy, and technology.

Professional development education needs development to assist L2 educators to combine technology into their education, instead of easily embedding technology into the current education and material area. Educational program planners, particularly the ones engaged in planning educator training programs, ought to offer a setting rich in technology for futuristic educators and engage them in tasks that assist them to expand techno pedagogical education content that will finally bring about improved learning results. EFL educators in China were found to be willing to apply technology and merge it successfully within the English curriculum and their routine class activity, leading to reciprocal advantages for both educators and learners. Consequently, it is suggested that policy-makers consider the demanding situations educators face after they employ technology to ease the technology merging process through presenting professional development education.

Even though educators held that employing technology improves class interplay with virtual native students, they could not choose the appropriate technologies that match pedagogy in the educational program in China. Consequently, the Education Ministry is advised to arrange education workshops wherein multiple apps are suggested to educators. Shortage of technology preparations, low-quality class infrastructure, and absence of technical aid in schools are difficulties that need to be solved by the Education Ministry. Professional development program designers and facilitators need to investigate the notion of essential professional development trying to strengthen English language educators to measure and sense with sufficient confidence to change their pedagogical activities and expert fields. Accordingly, technology merging was beneficial for educators learning to help to learn, as the task offered a chance for educators to use the new information in new unique conditions through designing certain concrete activities.

This study is a review-type article; furthermore, scholars attracted to examining technology merging in language education are also suggested to investigate more technology merging activities regarding learning tasks, designing activities, and case developing. As it is known that in improving chances of professional development, educators’ viewpoints and previous experiences with technology ought to be considered, extra research might be conducted to consider understanding regarding technology, viewpoints toward making use of technology, educators’ preparation, and computer accessibility, which all directly affect technology merging.

## Ethics Statement

The studies involving human participants were reviewed and approved by the Zhengzhou Business University Academic Ethics Committee. The patients/participants provided their written informed consent to participate in this study.

## Author Contributions

The author confirms being the sole contributor of this work and has approved it for publication.

## Conflict of Interest

The author declares that the research was conducted in the absence of any commercial or financial relationships that could be construed as a potential conflict of interest.

## Publisher’s Note

All claims expressed in this article are solely those of the authors and do not necessarily represent those of their affiliated organizations, or those of the publisher, the editors and the reviewers. Any product that may be evaluated in this article, or claim that may be made by its manufacturer, is not guaranteed or endorsed by the publisher.

## References

[B1] Adnan A. Ahmad M. Yusof A. Mohd Kamal M. Mustafa Kamal N. (2019). “English language simulations augmented with 360-degrees spherical videos: virtual reality’ real life learning!,” in *Proceedings of the International Invention, Innovative & Creative Conference: EFL Teachers’ Challenges In The Integration Of Technology For Online Classrooms During Coronavirus Pandemic In Iran*, (Senawang: MNNF Publisher),

[B2] Aghaei K. Ghoorchaei B. Rajabi M. Ayatollahi M. (2022). Iranian EFL learners’ narratives in a pandemic pedagogy: appreciative inquiry-based approach. *Lang. Relat. Res.* 13

[B3] Aghaei K. Rajabi M. Lie K. Y. Ajam F. (2020). Flipped learning as situated practice: a contrastive narrative inquiry in an EFL classroom. *Educ. Information Technol.* 25 1607–1623. 10.1007/s10639-019-10039-9

[B4] Ahmed P. S. (2012). The way we teach, the way they learn. *Proc. Soc. Behav. Sci.* 47 1554–1557. 10.1016/j.sbspro.2012.06.860

[B5] Albirini A. (2006). Cultural perceptions: the missing element in the implementation of ICT in developing countries. *Int. J. Educ. Dev. ICT* 2 49–65.

[B6] Alsied S. M. Pathan M. M. (2013). The use of computer technology in EFL classroom: advantages and implications. *IJ ELTS* 1 61–71.

[B7] Altun M. Khurshid Ahmad H. (2021). The use of technology in English language teaching: a literature review. *Int. J. Soc. Sci. Educ. Stud.* 8 226–232.

[B8] Ansyari M. F. (2015). Designing and evaluating a professional development program for basic technology integration in English as a foreign language (EFL) classrooms. *Australasian J. Educ. Technol.* 31 699–712. 10.14742/ajet.1675

[B9] Arnseth H. C. Hatlevik O. E. (2010). “Challenges in aligning pedagogical practices and pupils’ competencies with the Information Society’s demands: the case of Norway,” in *Cases On Interactive Technology Environments And Transnational Collaboration: Concerns And Perspectives*, eds Mukerji S. Triphati P. (Hershey: IGI Global), 266–280. 10.4018/978-1-61520-909-5.ch014

[B10] Avalos B. (2011). Teacher professional development in teaching and teacher education over ten years. *Teach.Teach. Educ.* 27 10–20. 10.1016/j.tate.2010.08.007

[B11] Bennett E. E. (2012). *A Four-Part Model Of Informal Learning: Extending Scharwenka’s Conceptual Model.* Available online at: http://www.adulterc.org/Proceedings/2012/papers/bennett [Accessed January 2022].

[B12] Brinkerhoff J. (2006). Effects of a long-duration, professional development academy on technology skills, computer self-efficacy, and technology integration beliefs and practices. *Int. Soc. Technol. Educ.* 39 22–43. 10.1080/15391523.2006.10782471

[B13] Cowan J. E. (2008). Strategies for planning technology enhanced learning experiences. *Clearing House* 82 55–59. 10.3200/TCHS.82.2.55-59

[B14] Craft A. (2000). *Continuing Professional Development.* London: Routledge and Falmer.

[B15] Crandall J. A. (2000). Language teacher education. *Annu. Rev. Appl. Linguistics* 20 34–55. 10.1017/S0267190500200032

[B16] Cuban L. (2009). *Oversold And Underused: Computers In The Classroom.* Cambridge, MA: Harvard University Press. 10.2307/j.ctvk12qnw

[B17] Culp K. M. Honey M. Mandinach E. (2005). A retrospective on twenty years of education technology policy. *J. Educ. Comput. Res.* 32 279–307. 10.2190/7W71-QVT2-PAP2-UDX7 22612255

[B18] Dockstader J. (2008). *Teachers of the 21st Century Know The What, Why, And How Of Technology Integration.*

[B19] Freeman D. (2004). “Implications of sociocultural perspectives for language teacher Education,” in *Language Learning And Teacher Education*, ed. Hawkins M. (Great Britain: Multilingual Matters Ltd), 169–197. 10.21832/9781853597657-010

[B20] Freeman L. D. Anderson M. (2011). *Techniques And Principles In Language Teaching.* Oxford: Oxford OUP.

[B21] Garet M. S. Porter A. C. Desimone L. Birman B. F. Yoon K. S. (2001). What makes professional development effective? Results from a national sample of teachers. *Am. Educ. Res. J.* 38 915–946. 10.3102/00028312038004915

[B22] Gee J. P. (2004). *Situated Language And Learning: A Critique Of Traditional Schooling.* New York, NY: Routledge.

[B23] Gençlter B. (2015). How does technology affect the language learning process at an early age? *Proc. Soc. Behav. Sci.* 199 311–316. 10.1016/j.sbspro.2015.07.552

[B24] Gilakjani A. P. (2017). A review of the literature on the integration of technology into the learning and teaching of English language skills. *Int. J. Engl. Linguistics* 7 95–106. 10.5539/ijel.v7n5p95

[B25] Gilakjani A. P. Sabouri N. B. (2014). Role of Iranian EFL teachers about using pronunciation power software in the instruction of English pronunciation. *Engl. Lang. Teach.* 7 139–148. 10.5539/elt.v7n2p20

[B26] Giordano V. (2008). A professional development model top promotes Internet integration into p-12 teachers’ practice: a mixed methods study. *Comput. Sch.* 24 111–123. 10.1300/J025v24n03_08

[B27] Günüç S. Kuzu A. (2014). Factors influencing student engagement and the role of technology in student engagement in higher education: campus-class-technology theory. *Turkish Online J. Qual. Inq.* 5 86–113. 10.17569/tojqi.44261

[B28] Hakim B. (2020). Technology integrated online classrooms and the challenges faced by the EFL teachers in Saudi Arabia during the COVID-19 pandemic. *Int. J. Appl. Linguist. Engl. Lit.* 9 33–39. 10.7575/aiac.ijalel.v.9n.5p.33

[B29] Harmer J. (2007). *The Practice Of English Language Teaching.* England: Pearson.

[B30] Hartono R. (2016). *Indonesian EFL Teachers’ Perceptions And Experiences of Professional Development*. Ph.D. thesis. Indiana, PA: Indiana University of Pennsylvania.

[B31] Hough B. W. Smithey M. W. Evertson C. M. (2004). Using computer mediated communication to createvirtual communities of practice for intern teachers. *J. Technol. Teach. Educ.* 12 361–386.

[B32] Isman A. (2012). Technology and technique: an educational perspective. *TOJET* 11 207–213.

[B33] Jabbari N. Boriack A. W. Barohona E. Padrón Y. N. Waxman H. C. (2017). “Social networking,” in *The TESOL Encyclopedia of English Language Teaching*, ed. Liontas J. I. (Alexandria, VA: TESOL International Association), 1–7. 10.1002/9781118784235.eelt0430

[B34] Jiang L. Zhang L. J. May S. (2019). Implementing English-medium instruction (EMI) in China: teachers’ practices and perceptions, and students’ learning motivation and needs. *Int. J. Biling. Educ. Biling.* 22 107–119. 10.1080/13670050.2016.1231166

[B35] Johnston B. (2009). “Collaborative teacher development,” in *The Cambridge Guide To Language Teacher Education*, eds Burns A. Richards J. C. (Cambridge: Cambridge University Press), 241–229.

[B36] Kao C. P. Tsai C. C. Shih M. (2014). Development of a survey to measure self-efficacy and attitudes toward web-based professional development among elementary school teachers. *J. Educ. Technol. Soc.* 17 302–315. 10.1037/t59863-000

[B37] Krolak-Schwerdt S. Glock S. Bohmer M. (2014). *Teachers’ Professional Development: Assessment, Training, And Learning.* Rotterdam: SensePublishers. 10.1007/978-94-6209-536-6

[B38] Lavonen J. Juuti K. Aksela M. Meisalo V. (2006). A professional development project for improving the use of information and communications technologies in science teaching. *Technol. Pedagogy and Educ.* 15 159–174. 10.1080/14759390600769144

[B39] Lawless K. A. Pellegrino J. W. (2007). Professional development in integrating technology into teaching and learning: knowns, unknowns, and ways to pursue better questions and answers. *Rev. Educ. Res.* 77 575–615. 10.3102/0034654307309921

[B40] Lee H. (2014). *The Intersection Between Professional Development and Professional Learning Communities: Working Towards Improving the Educational Experiences of English Learners*. Doctoral Dissertation. Baltimore, MD: University of Maryland, Baltimore County.

[B41] Lee K. W. James C. C. (2018). Exploring a transformative teacher professional development model to engender technology integration in the 21st century ESL language classrooms. *Int. J. Comput. Assist. Lang. Learn. Teach.* 8 13–31. 10.4018/IJCALLT.2018100102

[B42] Lin L. C. (2009). An integrated framework for the development of radio frequency identification technology in the logistics and supply chain management. *Comput. Ind. Eng.* 57 832–842. 10.1016/j.cie.2009.02.010

[B43] Margolis J. Durbin R. Doring A. (2017). The missing link in teacher professional development: student presence. *Prof. Dev. Educ.* 43 23–35. 10.1080/19415257.2016.1146995

[B44] Martins J. Gonçalves R. Oliveira T. Cota M. Branco F. (2016). Understanding the determinants of social network sites adoption at firm level: a mixed methodology approach. *Electronic Commerce Res. Appl.* 18 10–26. 10.1016/j.elerap.2016.05.002

[B45] Mizell H. (2010). *Why Professional Development Matters?.* Oxford: Oxford Learning Forward.

[B46] Murray A. (2010). Empowering teachers through professional development. *Engl. Teach. Forum* 48 2–11.

[B47] Nicholas H. Ng W. (2012). Factors influencing the uptake of a mechatronics curriculum initiative in five Australian secondary schools. *Int. J. Technol. Design Educ.* 22 65–90. 10.1007/s10798-011-9156-6

[B48] Patil D. P. (2020). Trends and challenges in english language teaching. *Stud. Indian Place Names* 40 158–164.

[B49] Penuel W. (2006). Implementation and effects of one-to-one computing initiatives: a research synthesis. *J. Res. Technol. Educ.* 38 329–348. 10.1080/15391523.2006.10782463

[B50] Prensky M. (2008). The role of technology. *Educ. Technol.* 48 1–3.

[B51] Richards J. C. (2014). “Foreword,” in *English As A Foreign Language Teacher Education: Current Perspectives And Challenges*, ed. Agudo J. D. D. M. (Amsterdam: Rodopi), 1–3. 10.1163/9789401210485_002

[B52] Rodinadze S. Zarbazoia K. (2012). The advantages of information technology in teaching English language. *Front. Lang. Teach.* 3:271–275.

[B53] Sabzian F. Gilakjani A. P. Sodouri S. (2013). Use of technology in classroom for professional development. *J. Lang. Teach. Res.* 4 684–692. 10.4304/jltr.4.4.684-692

[B54] Shyamlee S. D. Phil M. (2012). Use of technology in English language teaching and learning: an analysis. *Int. Conf. Lang. Med. Cult.* 33 150–156.

[B55] Tajeddin Z. Rezanejad A. (2019). Intercultural teaching in L2 classrooms: exploring English language teachers’ beliefs. *Teach. Engl. Foreign Lang.* 1 12–15.

[B56] Timucin M. (2006). Implementing call in the EFL context. *ELT J.* 60 262–271. 10.1093/elt/ccl006

[B57] Wang Y. L. (2017). Construction elements and path of practical education model in universities. *EURASIA J. Math. Sci. Technol.* 13 6775–6782. 10.12973/ejmste/78525

[B58] Wenger E. White N. Smith J. D. (2009). *Digital Habitats: Stewarding Technology For Communities.* Portland, OR: CPsquare.

[B59] Wilden E. Porsch R. (2017). “Researching the professional development of primary EFL teachers. An introduction,” in *The Professional Development Of Primary EFL Teachers: National And International Research*, eds Wilden E. Porsch R. (Münster: Waxmann), 7–23.

[B60] Wong M. S. (2011). Fifty ways to develop professionally: what language educators need to succeed. *Lang. Educ. Asia* 2 142–155. 10.5746/LEiA/11/V2/I1/A12/Wong

[B61] Yadav N. Gupta K. Khetrapal V. (2018). Next education: technology transforming education. *South Asian J. Bus. Manage. Cases* 14 68–77. 10.1177/2277977918754443

[B62] Young S. C. (2003). Integrating ICT into second language education in a vocational high school. *J. Comput. Assist. Learn.* 19 447–461. 10.1046/j.0266-4909.2003.00049.x

[B63] Yunus M. M. (2007). Malaysian ESL teachers’ use of ICT in their classrooms: expectations and realities. *Recall* 9 79–95. 10.1017/S0958344007000614

[B64] Yurdakul B. Yıldız D. Çakar E. Uslu Ö (2010). “Evaluation of professional development program practices implemented toward web-based content development,” in *Proceedings 13th International Conference ICT in the education of the Balkan countries*, Varna.

